# A generic ^89^Zr labeling method to quantify the in vivo pharmacokinetics of liposomal nanoparticles with positron emission tomography

**DOI:** 10.2147/IJN.S134379

**Published:** 2017-04-20

**Authors:** Nan Li, Zilin Yu, Truc Thuy Pham, Philip J Blower, Ran Yan

**Affiliations:** 1 Division of Imaging Sciences and Biomedical Engineering, St Thomas’ Hospital, King’s College London, London, UK; 2 Tianjin Key Laboratory for Modern Drug Delivery & High-Efficiency, School of Pharmaceutical Science and Technology, Tianjin University, Tianjin, People’s Republic of China

**Keywords:** liposome, zirconium-89, PET, pharmacokinetics

## Abstract

Liposomal nanoparticles are versatile drug delivery vehicles that show great promise in cancer therapy. In an effort to quantitatively measure their in vivo pharmacokinetics, we developed a highly efficient ^89^Zr liposome-labeling method based on a rapid ligand exchange reaction between the membrane-permeable ^89^Zr(8-hydroxyquinolinate)_4_ complex and the hydrophilic liposomal cavity-encapsulated deferoxamine (DFO). This novel ^89^Zr-labeling strategy allowed us to prepare radiolabeled forms of a folic acid (FA)-decorated active targeting ^89^Zr-FA-DFO-liposome, a thermosensitive ^89^Zr-DFO-liposome, and a renal avid ^89^Zr-PEG-DFO-liposome at room temperature with near-quantitative isolated radiochemical yields of 98%±1% (n=6), 98%±2% (n=5), and 97%±1% (n=3), respectively. These ^89^Zr-labeled liposomal nanoparticles showed remarkable stability in phosphate-buffered saline and serum at 37°C without leakage of radioactivity for 48 h. The uptake of ^89^Zr-FA-DFO-liposome by the folate receptor-overexpressing KB cells was almost 15-fold higher than the ^89^Zr-DFO-liposome in vitro. Positron emission tomography imaging and ex vivo biodistribution studies enabled us to observe the heterogeneous distribution of the ^89^Zr-FA-DFO-liposome and ^89^Zr-DFO-liposome in the KB tumor xenografts, the extensive kidney accumulation of the ^89^Zr-FA-DFO-liposome and ^89^Zr-PEG-DFO-liposome, and the different metabolic fate of the free and liposome-encapsulated ^89^Zr-DFO. It also unveiled the poor resistance of all three liposomes against endothelial uptake resulting in their catabolism and high uptake of free ^89^Zr in the skeleton. Thus, this technically simple ^89^Zr-labeling method would find widespread use to guide the development and clinical applications of novel liposomal nanomedicines.

## Introduction

Liposomal nanoparticles are versatile drug delivery systems that can treat malignant tumors by combining the strengths of various therapeutic regimens such as chemo-, thermo-, and phototherapy.^1^ Thermosensitive liposomes releasing encapsulated drugs under mild hyperthermia (<45°C)^2^ and active targeting liposomes decorated with cancer-specific ligands,^3^ with their ability of selective drug delivery to the tumor sites, have shown great promise in cancer treatment. However, the potential therapeutic efficacy of these nanomedicines can vary greatly among patients because of the tumor heterogeneity and variable vascular permeability. To provide personalized cancer treatment, it would be immensely beneficial to screen liposomal tumor uptake on a patient-to-patient basis prior to therapy.^4^

PET is a noninvasive nuclear imaging technique that can be used to obtain quantitative measurement of the pharmacokinetic profile of the radiolabeled liposomes in real time.^5^ Fluorine-18 (t_1/2_ =110 min)^6^ and copper-64 (t_1/2_ =12.7 h)^7^ have been incorporated into liposomes. However, due to the long circulation half-lives of liposomal nanoparticles (typically in the order of days), it is essential to select longer half-life radioisotopes such as zirconium-89 (t_1/2_ =3.3 d) to gain the full picture of their pharmacokinetics with PET. Current labeling methods are based on the conjugation of ^89^Zr to the liposomal surface. Abou et al reported that ^89^Zr can be directly adsorbed on the lipid membrane through interaction between the metal cation and phospholipid phosphate anion.^8^ This method requires elevated temperature at 45°C to achieve effective radiolabeling (99%). Due to the weak binding affinity between the ^89^Zr and membrane phospholipid, 60% or 13%, respectively, of ^89^Zr was washed off from the liposome when challenged with either ethylenediaminetetraacetic acid (5 mM) or PBS in vitro. Seo et al coupled the strong zirconium chelator, DFO, with PEG1k-DSPE lipid. They then incorporated the corresponding DFO-PEG1k-DSPE lipid into DFO-PEG1k liposome.^9^ Moderate but variable RCYs of 68%±24% (n=9) were obtained at room temperature. Pérez-Medina et al employed a copper-free click reaction to label ^89^Zr with a DFO azide and then couple the corresponding ^89^Zr-DFO azide to the lipid-bonded dibenzocyclooctynyl group on the liposome surface.^10^ Low isolated RCY of 14% was observed in this two-step method with a prolonged preparation time of 16 h. As an alternative to the lipid membrane-labeling strategy, the radioisotopes can also be localized in the liposomal cavity. In 2011, Petersen et al reported a remote loading approach to label liposomes with ^64^Cu.^7^ It utilizes a lipophilic weak copper ligand, 2-HQ, to transport ^64^Cu across the liposomal membrane for ligand exchange with a hydrophilic stronger copper ligand, 1,4,7,10-tetraazacyclododecane-1,4,7,10-tetraacetic acid (DOTA). Excellent RCYs (95.5%±1.6%, n=11) were obtained. Recently, Ferris et al demonstrated that a lipophilic [^89^Zr]oxinate_4_ complex can be used for cell labeling by crossing the cell membrane and reacting with intracellular species to deposit ^89^Zr in the cell.^11^,^12^ Edmonds et al used oxinate complexes in a similar approach to label liposomes containing anticancer drugs with the capability to chelate metal ions with ^64^Cu, ^89^Zr, and ^52^Mn.^13^ They used an elevated temperature of 50°C to achieve effective ^89^Zr liposome radiolabeling. These “ionophore”-based methods open up the opportunity to radiolabel liposomal nanoparticles by encapsulating ^89^Zr in their aqueous cavity.

**Figure 1:**
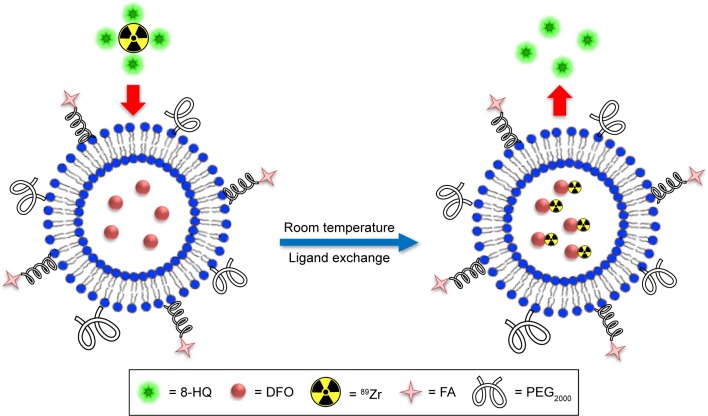
^89^Zr labeling of an FA-decorated FA-DFO-liposome through a room-temperature ligand exchange reaction between the ^89^Zr(8-HQ)_4_ complex and the encapsulated DFO in the liposomal aqueous cavity. **Abbreviations:** FA, folic acid; DFO, deferoxamine; 8-HQ, 8-hydroxyquinoline; PEG_2000_, (polyethylene glycol)-2000.

For a generic strategy to radiolabel liposomal nanoparticles, especially thermosensitive liposomes, with ^89^Zr, it is critical for the radiochemical reaction to take place at room temperature with reproducibly high isolated RCYs (to avoid the need for a post-labeling purification step) and irrespective of the nature of the loaded drugs. The labeled liposomes should also have prolonged radiolabel retention after formulation. Herein, we describe a technically simple ^89^Zr-labeling method suitable for labeling a variety of liposomal nanoparticles with near-quantitative isolated RCYs at room temperature. The strategy employs 8-HQ (oxine) to deliver ^89^Zr into the liposomal cavity for ligand exchange with encapsulated DFO (Figure 1). We also demonstrated the application of PET imaging in the quantitative measurement of the in vivo pharmacokinetics of three different ^89^Zr-labeled liposome nanoparticles.

## Materials and methods

### Reagents

DSPE-PEG_2000_ and DPPC were purchased from NOF Europe (Frankfurt, Germany). DSPE-PEG_2000_ Folate (ammonium salt) was purchased from Stratech Scientific Ltd. All other reagents were purchased from Sigma-Aldrich and used without further purification.

### Preparation and characterization of DFO-encapsulated liposomes

All liposomes were prepared using well-established literature methods.^14^–^16^ For FA-DFO-liposome, DPPC (21.3 mg, 29 µmol), DSPE-PEG_2000_ (2.7 mg, 1.0 µmol), DSPE-PEG_2000_ Folate (1.0 mg, 0.3 µmol), and cholesterol (5.0 mg, 13 µmol) were dissolved in methanol (4.0 mL) and sonicated for 2 min in a round-bottom flask. Then, chloroform (20 mL) was added into the above solution and sonicated for another 2 min. The solution was heated at 50°C under vacuum in a rotary evaporator to remove all the solvents and form a thin lipid film. Subsequently, the DFO (mesylate salt) water solution (5 mL, 10 mM, pH 7.0) was added and incubated at 35°C for 30 min and then sonicated for another 5 min. In order to remove the unencapsulated DFO salt from the FA-DFO-liposome, the above dispersion was dialyzed against deionized water using a 6 kDa cut-off membrane. The deionized water was changed three times in 48 h, and the presence of DFO was examined by mass spectrometry. The FA-DFO-liposome was generated by sequential extrusions through 800, 400, 200, and 100 nm polycarbonate filters (five times each), and stored at 4°C for further use. The DFO-liposome and PEG-DFO-liposome were prepared following the same protocol. For DFO-liposome, DPPC (21.3 mg, 29 µmol), DSPE-PEG_2000_ (3.7 mg, 1.3 µmol), and cholesterol (5.0 mg, 13 µmol) were used. For PEG-DFO-liposome, DPPC (16.0 mg, 22 µmol), DSPE-PEG_2000_ (9.0 mg, 3 µmol), and cholesterol (4.0 mg, 10 µmol) were used. The mean diameter, particle size distribution, and zeta potential were measured using a Malvern Zetasizer Nano (Malvern Instruments Ltd., Malvern, UK). The morphology of DFO-liposomes, PEG-DFO-liposomes, and FA-DFO-liposomes was analyzed by TEM. A droplet of particle dispersion with a 1:10 dilution was placed on a carbon-coated copper grid, and then developed into a thin liquid film. Samples were stained negatively by the 0.5% (w/v) solution of phosphotungstic acid. The excess solution was removed by a filter paper. Then samples were completely air-dried. Images were obtained with the JEM-100 CX (Jeol Ltd., Tokyo, Japan) transmission electron microscope at a 190,000× magnification and an accelerating voltage of 80 kV.

### Radiochemistry

^89^Zr was supplied as Zr^4+^ in 1.0 M oxalic acid (PerkinElmer, Waltham, MA, USA). It was neutralized to pH 7.0 with HEPES buffer (1.0 M, pH 7.0) for radiolabeling.

### Chelation of ^89^Zr with 8-HQ

^89^Zr (5.0 MBq) in HEPES buffer (100 µL, 1.0 M, pH 7.0) was added to the freshly prepared 8-HQ (23 µg, 0.16 µmol) in HEPES buffer (50 µL, 10 mM, pH 7.0). The resulting solution was kept at room temperature for 30 min. The reaction was then analyzed by an Agilent 1200 HPLC system equipped with a series diode array detector and Raytest GABI star radioactivity detector. A ZORBAX HPLC column (300SB-C18, 9.4×250 mm, 5 µm) with an eluent of MeOH/H_2_O (0.1% TFA) and a flow rate of 2.5 mL/min was used following the gradient: 5% MeOH 0–5 min; 5%–90% MeOH 5–18 min; 90% MeOH 18–25 min; and 90%–5% MeOH 25–30 min. The HPLC retention time for ^89^Zr(8-HQ)_4_ was 16.6 min, whereas the retention time for free ^89^Zr was 3.5 min (Figure S1). The radiolabeling was also monitored by silica gel 60 radioTLC (Merck) with DTPA (50 mM, pH 7.0) as the mobile phase. ^89^Zr(8-HQ)_4_ had an Rf =0.6 with streaking, whereas ^89^Zr from pH 7.0 HEPES buffer had an Rf =0.9 with streaking.

### Chelation of ^89^Zr with DFO

^89^Zr (5.0 MBq) in HEPES buffer (100 µL, 1.0 M, pH 7.0) was added to DFO (100 µL, 20 mM, pH 7.0) in water. The resulting solution was kept at room temperature for 60 min before HPLC analysis using a ZORBAX HPLC column (300SB-C18, 9.4×250 mm, 5 µm) with an eluent of MeOH/H_2_O (0.1% TFA) and a flow rate of 2.5 mL/min. The following gradient was used: 5% MeOH 0–5 min; 5%–90% MeOH 5–18 min; 90% MeOH 18–25 min; and 90%–5% MeOH 25–30 min. The HPLC retention time for ^89^Zr-DFO was 16.4 min, whereas the retention time for free ^89^Zr was 3.5 min (Figure S2). The radiolabeling was also monitored by silica gel 60 radioTLC (Merck) with DTPA (50 mM, pH 7.0) as the mobile phase. ^89^Zr-DFO had an Rf =0.0, whereas ^89^Zr from pH 7.0 HEPES buffer had an Rf =0.9 with streaking.

### Ligand exchange between ^89^Zr(8-HQ)_4_ and DFO

^89^Zr (5.0 MBq) in HEPES buffer (100 µL, 1.0 M, pH 7.0) was added to the freshly prepared 8-HQ (23 µg, 0.16 µmol) in HEPES buffer (50 µL, 10 mM, pH 7.0). The resulting solution was kept at room temperature for 30 min before addition of DFO (150 µL, 20 mM, pH 7.0) in water. This solution was kept at room temperature for another 60 min. The radiolabeling efficiency was monitored by silica gel 60 radioTLC (Merck) with DTPA (50 mM, pH 7.0) as the mobile phase. ^89^Zr-DFO had an Rf =0.0 and ^89^Zr(8-HQ)_4_ had an Rf =0.6 with streaking, whereas ^89^Zr from pH 7.0 HEPES buffer had an Rf =0.9 with streaking.

### ^89^Zr labeling of liposomes

^89^Zr (5.0 MBq) in HEPES buffer (100 µL, 1.0 M, pH 7.0) was added to the freshly prepared 8-HQ (23 µg, 0.16 µmol) in HEPES buffer (50 µL, 10 mM, pH 7.0). The resulting solution was kept at room temperature for 30 min before addition of a DFO-liposome solution (300 µL, approximately 1.8 µmol of lipids). The reaction mixture was kept at room temperature for another 60 min. The ^89^Zr-DFO-liposome was purified with a PD MiniTrap G 25 size exclusion column (GE Healthcare) eluting with PBS buffer following the manufacturer’s instructions. The ^89^Zr-DFO-liposome was distributed in the fraction from 0.4 to 2.2 mL (Figure S3). The free ^89^Zr, ^89^Zr(8-HQ), and ^89 4^ Zr-DFO were retained on the column until the eluting volume reached 4.4 mL. The ^89^Zr-FA-DFO-liposome and ^89^Zr-PEG-DFO-liposome were prepared and purified using the same protocol.

### Stability study of ^89^Zr-labeled liposomes

The purified ^89^Zr-FA-DFO-liposome (10 MBq) in PBS was either stored at 4°C or incubated at 37°C for 48 h. An aliquot (5 MBq) was taken and purified by a PD MiniTrap G 25 column (GE Healthcare) eluting with PBS at 24 and 48 h to monitor the radiolabel retention. The stability of both ^89^Zr-DFO-liposome and ^89^Zr-PEG-DFO-liposome in PBS at 37°C for 48 h was also determined following the same procedure. The stability of ^89^Zr-FA-DFO-liposome in fresh rat serum at 37°C for 24 and 48 h was also examined by mixing rat serum and ^89^Zr-FA-DFO-liposome in PBS (1:1) and following the same procedure. The release of radioactivity from the thermosensitive ^89^Zr-DFO-liposome at mild hyperthermia of 45°C in PBS at 24 and 48 h was also determined following the same procedure.

### In vitro KB cell uptake of ^89^Zr-FA-DFO-liposome and ^89^Zr-DFO-liposome

The KB cells (ATCC^®^ CCL-17™) were cultivated at 37°C in 5% CO_2_ atmosphere in the Dulbecco’s Modified Eagle’s Medium supplemented with 10% fetal bovine serum, 200 U/L penicillin, 0.1 g/L streptomycin, and 2 mM l-glutamine. KB cells were plated on a six-well plate and incubated in the cell culture media for 16 h. Once confluent, ^89^Zr-FA-DFO-liposome was added to three wells, while ^89^Zr-DFO-liposome was added to the remaining three wells. The cells were incubated at 37°C for 1 h before removal of the media and washed three times with PBS. The KB cells were trypsinized and suspended in PBS. The combined cell media with PBS wash and the cell suspensions were gamma-counted using an LKB Wallac 1282 compugamma CS Universal Gamma Counter. The number of cells in each well was determined with a hemocytometer.

### KB tumor xenografts development

All animal experiments complied with the Animals (Scientific Procedures) Act (UK 1986) and Home Office (UK) guidelines and were conducted under a Home Office licence with local ethical approval by the KCL College Research Ethics Committee (CREC). Four-week-old female CD-1/nu/nu mice (Charles River UK Ltd.) were subcutaneously injected with 1.0×10^6^ KB cells in 50 µL of saline in the front-right flank. Tumor volume was estimated using the formula V = (L * W * H)/2.

### PET/CT imaging and data analysis

Preclinical PET/CT images were acquired using a NanoScan^®^ PET/CT (Mediso Medical Imaging Systems, Budapest, Hungary) scanner with mice under isoflurane (2% in oxygen) anesthesia. The KB tumor xenograft-bearing CD1 nude mice (n=3 for each liposome) each received approximately 5.0 MBq of either ^89^Zr-FA-DFO-liposome or ^89^Zr-DFO-liposome in 100 µL PBS via i.v. injection. PET scanning was performed for 30 min at 6, 24, and 48 h postinjection followed by a CT scan. All PET/CT data were reconstructed with the Monte Carlo-based full-three-dimensional iterative algorithm Tera-Tomo (Mediso Medical Imaging Systems). Raw PET data were reconstructed into 30-min bins using reconstruction settings (four iterations, six subsets, 0.4×0.4×0.4 mm^3^ voxel size) as well as intercrystal scatter correction. All reconstructed data were analyzed with VivoQuant software (v2.5; inviCRO, LLC, Boston, MA, USA).

### Ex vivo biodistribution studies

KB tumor xenograft-bearing CD1 nude mice (n=3) that had received ^89^Zr-DFO (0.5 MBq) in 100 µL PBS via i.v. injection, and healthy CD1 mice (n=3) that had received ^89^Zr-PEG-DFO-liposome (0.5 MBq) in 100 µL PBS via i.v. injection were culled by cervical dislocation 48 h postinjection. Tumor xenograft, major thoracoabdominal organs, the left femur, and thigh muscle were harvested, weighed, and gamma-counted. The distribution of ^89^Zr in each organ was expressed as % ID/g. The total injected dose was defined as the sum of the whole-body counts excluding the tail. Tissues from KB tumor xenograft-bearing CD1 nude mice at the end of above PET/CT scanning experiments (48 h postinjection) were analyzed similarly.

## Results

### Optimizing the preparation of ^89^Zr(8-HQ)_4_ and ^89^Zr-DFO, and ligand exchange between ^89^Zr(8-HQ)_4_ and DFO

The ^89^Zr(8-HQ)_4_ was formed in near-quantitative RCY as determined by radioTLC and HPLC (Figure S1) in 30 min at room temperature by mixing 8-HQ (1.1 mM) and ^89^Zr-oxalate in pH 7.0 HEPES buffer. The labeling efficiencies for DFO at three concentrations (1.0, 10.0, and 100.0 mM) with ^89^Zr-oxalate in pH 7.0 HEPES buffer at room temperature in 60 min were 32%, 100%, and 100%, respectively, as measured by radioTLC and HPLC (Figure S2). Next, the ligand exchange reactions between ^89^Zr(8-HQ)_4_ and DFO were carried out by sequential incubation of ^89^Zr with 8-HQ (1.1 mM) for 30 min before addition of DFO in water to reach the final DFO concentration of either 10.0 or 100.0 mM. ^89^Zr(8-HQ)_4_ was quantitatively converted to ^89^Zr-DFO at room temperature within 60 min under both conditions as determined by radioTLC (Figure S3 presents the representative radioTLC of free ^89^Zr, ^89^ZrDFO, and ^89^Zr(8-HQ)_4_). Thus, 10.0 mM of DFO was chosen for incorporation within the liposomes to irreversibly trap the ^89^Zr.

### Liposome preparation and characterization

**Figure 2:**
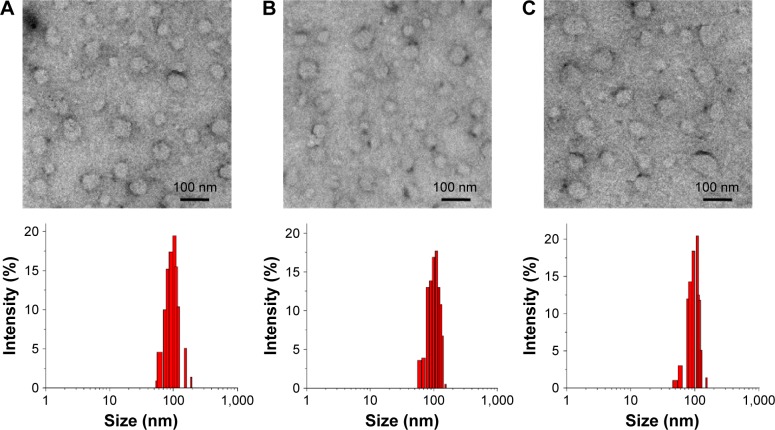
TEM images and particle size distributions of (**A**) DFO-liposome, (**B**) FA-DFO-liposome, and (**C**) PEG-DFO-liposome. **Abbreviations:** TEM, transmission electron microscopy; DFO, deferoxamine; FA, folic acid; PEG, polyethylene glycol.

DFO (10.0 mM) was encapsulated into the aqueous cavity of three different liposomal nanoparticles (a thermosensitive DFO-liposome, an FA-decorated active targeting FA-DFO-liposome, and a PEG-DFO-liposome containing three-fold higher PEG_2000_ in its membrane, compared to the other two liposomes) using the thin-layer hydration method. The unencapsulated DFO was removed by dialysis of the liposome dispersion using a 6 kDa cut-off membrane against deionized water for 48 h until no DFO was detected by mass spectrometry in the dialysate. The liposomal nanoparticles were then obtained by sequential extrusions through 800, 400, 200, and 100 nm polycarbonate filters. To characterize these new liposomal nanoparticles, their morphology was detected by TEM. These particles showed a regular circular shape without obvious differences among the three formulations (Figure 2). Furthermore, all of the liposomes showed good homogeneity with a PDI around 0.110–0.126, average particle sizes between 99.8 and 102.9 nm, and zeta potential ranging from −20.1 to −23.1 mV (Table S1).

### ^89^Zr liposome-labeling method development

To identify the optimal liposome-radiolabeling conditions, initially, ^89^Zr^4+^ in 1.0 M oxalic acid was neutralized to pH 7.0 with HEPES buffer (1.0 M, pH 7.0). An aliquot (5.0 MBq, 100 µL) was reacted with 8-HQ (50 µL) in pH 7.0 HEPES buffer at room temperature for 30 min to form the ^89^Zr(8-HQ)_4_. DFO-liposome water solution (150 µL, approximately 0.9 µmol of lipids) was then added resulting in a mixture with the formulation of ^89^Zr:8-HQ:DFO-liposome in a volume ratio of 2:1:3. After incubation for a further 60 min at room temperature, the reaction mixture was purified using a PD MiniTrap G 25 (GE Healthcare) size exclusion column. The ^89^Zr-DFO-liposome was isolated in near-quantitative RCYs of 98%±2% (n=3) (Table 1, entry 1). However, when liposomes labeled in this way were stored at 4°C for 48 h, the radiolabel retention of the ^89^Zr-DFO-liposome was reduced to 83%. The subsequent radioTLC analysis identified that the leaked radioactive material was ^89^Zr(8-HQ)_4_. To further confirm that the unreacted ^89^Zr(8-HQ)_4_ was the cause of radiolabel leakage, ^89^Zr(8-HQ)_4_ was incubated with a DFO-free blank liposome which was prepared with the same formulation as the DFO-liposome but only contained water in its aqueous cavity using the ^89^Zr:8-HQ:liposome (2:1:3) formulation under the same conditions. The radiolabeled liposome was obtained in a low isolated RCY of 22% (Table 1, entry 5). When this purified ^89^Zr-labeled liposome was stored at 4°C in PBS, the radioactivity retained in the liposome was reduced to 79% within 48 h indicating that the lipophilic ^89^Zr(8-HQ)_4_ can slowly leak out of the liposome after loading. Moreover, when this ^89^Zr-labeled blank liposome was incubated with DFO (10 mM) in PBS at room temperature for 60 min, there was no radioactivity in the liposome fractions after the size exclusion purification. All the radioactive material isolated was ^89^Zr-DFO as determined by both radioTLC and HPLC. To drive the ligand exchange reaction between ^89^Zr(8-HQ)_4_ and the liposome-encapsulated DFO to completion, we doubled the amount of DFO-liposome (300 µL, approximately 1.8 µmol of lipids) for the radiolabeling reaction resulting in a formulation of ^89^Zr:8-HQ:DFO-liposome in the volume ratio of 2:1:6. After size exclusion purification (Figure S4 shows a typical profile), the ^89^Zr-DFO-liposome was obtained with isolated RCYs of 98%±2% (n=5) (Table 1, entry 2). Using this new formulation, there was no measurable radiolabel leakage from the purified liposome when stored at 4°C for 48 h. To ensure that there was no membrane-bound ^89^Zr in the labeled liposome, the purified ^89^Zr-DFO-liposome was incubated with DFO (10 mM) in PBS at room temperature for 60 min and then passed through a size exclusion column. The ^89^Zr-DFO-liposome was quantitatively recovered from this DFO challenge experiment. Consequently, the optimized formulation of ^89^Zr:8-HQ:DFO-liposome in the volume ratio of 2:1:6 containing approximately 4.0 mM lipids was used for the radiolabeling of the FA-DFO-liposome and the PEG-DFO-liposome. Near-quantitative isolated RCYs of 98%±1% (n=6) and 97%±1% (n=3), respectively, were obtained (Table 1, entries 3 and 4). Other labeling conditions such as the absence of the lipophilic ligand (8-HQ) or use of 2-HQ as the ^89^Zr transporter were also investigated. Lower isolated RCYs around 81%±11% (n=3) and 83%, respectively, were observed (Table 1, entries 6 and 7).

### In vitro stability of the ^89^Zr-labeled liposomal nanoparticles

The purified ^89^Zr-FA-DFO-liposome was incubated either at 4°C or at 37°C in PBS for 48 h. The radiolabel retention at 24 and 48 h was monitored by size exclusion chromatography. The ^89^Zr-FA-DFO-liposome was quantitatively recovered at both time points and temperatures. Similarly, both the ^89^Zr-DFO-liposome and ^89^Zr-PEG-DFO-liposome quantitatively retained their radioactivity at 37°C in PBS for 48 h. In addition, the stability of ^89^Zr-FA-DFO-liposome in fresh rat serum at 37°C for 24 and 48 h was also examined, and its radiolabel retention was 95% and 94%, respectively. To demonstrate the release of the encapsulated radioactivity under mild hyperthermia, the thermosensitive ^89^Zr-DFO-liposome was heated at 45°C in PBS for 48 h. The radioactivity release was about 17%±3% and 26%±2% (n=3) at 24 and 48 h, respectively, as determined by size exclusion chromatography. HPLC analysis indicated that the released radioactive material was ^89^Zr-DFO.

### In vitro KB cell uptake study

To illustrate the targeting effect of the FA-decorated ^89^Zr-FA-DFO-liposome, the folate receptor-overexpressing KB cells were incubated with either the ^89^Zr-FA-DFO-liposome or ^89^Zr-DFO-liposome in cell culture media at 37°C for 1 h. The KB cell uptake of each liposome was 7.0%±1% and 0.47%±0.1% (n=3) incubation dose per million cells, respectively (Figure S5).

### PET imaging and biodistribution study

**Figure 3:**
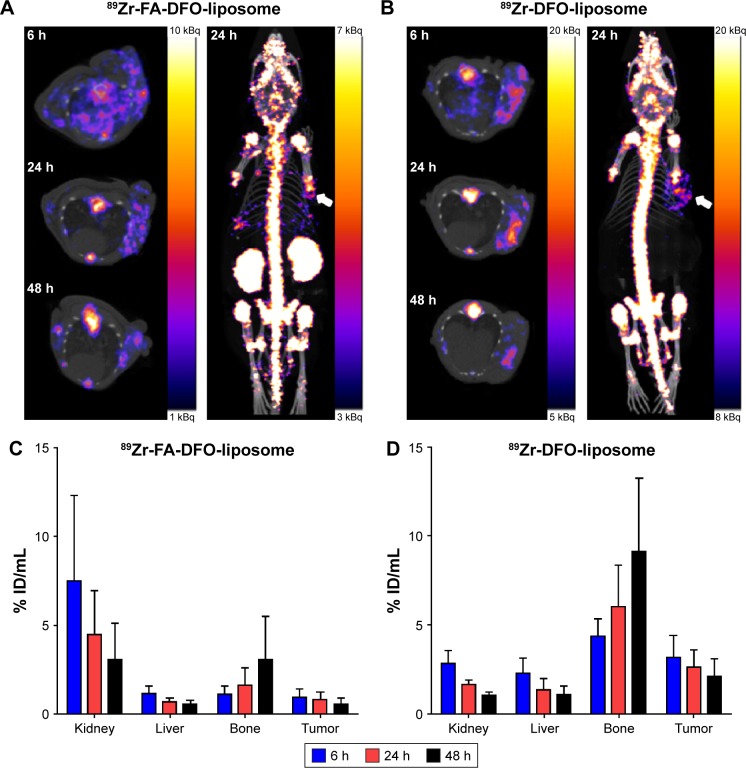
(**A** and **B**) Sequential axial PET images at 6, 24, and 48 h and three-dimensional PET images at 24 h post-i.v. injection of two KB tumor xenograft-bearing (in the front-right flank indicated by white arrows) CD1 nude mice that received either the ^89^Zr-FA-DFO-liposome or the ^89^Zr-DFO-liposome. (**C** and **D**) Radioactivity accumulation in selected organs extracted from the corresponding ^89^Zr-FA-DFO-liposome or ^89^Zr-DFO-liposome PET scans. **Abbreviations:** PET, positron emission tomography; i.v., intravenous; FA, folic acid; DFO, deferoxamine; PEG, polyethylene glycol; ID, injected dose.

**Figure 4:**
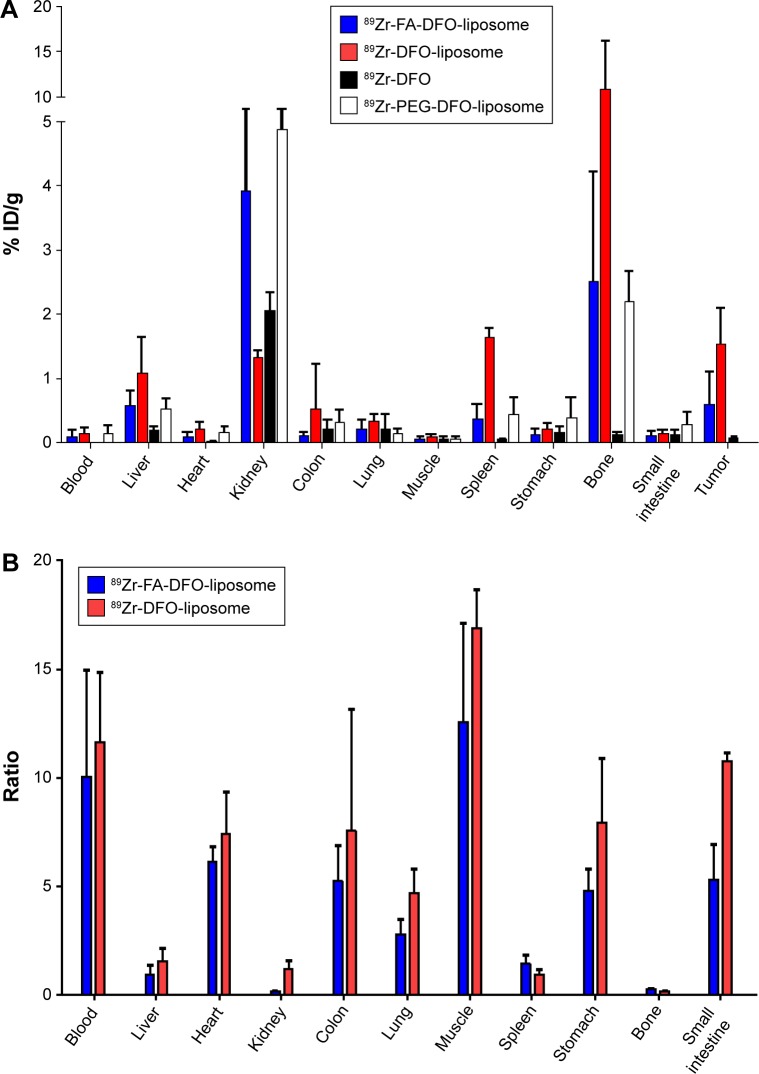
(**A**) Ex vivo biodistribution of the ^89^Zr-FA-DFO-liposome, ^89^Zr-DFO-liposome, and ^89^Zr-DFO in KB tumor xenograft-bearing CD1 nude mice (n=3) and ^89^Zr-PEG-FA-DFO-liposome in healthy CD1 mice (n=3) at 48 h post-i.v. injection. (**B**) Tumor-to-organ ratio of ^89^Zr-FA-DFO-liposome and ^89^Zr-DFO-liposome at 48 h post-i.v. injection. **Abbreviations:** FA, folic acid; DFO, deferoxamine; PEG, polyethylene glycol; i.v., intravenous; ID, injected dose.

Either ^89^Zr-FA-DFO-liposome or ^89^Zr-DFO-liposome (5 MBq) was administered intravenously to the KB tumor xenograft-bearing (in the front-right flank) CD1 nude mice (n=3) for three sequential PET/CT scans at 6, 24, and 48 h post-i.v. injection. The accumulation of both ^89^Zr-FA-DFO-liposome and ^89^Zr-DFO-liposome in the KB tumor xenografts was visualized at all three time points (Figure 3A and B). Moreover, the radioactivity signals were unevenly distributed within the tumor xenografts in both cases. To further analyze the in vivo kinetics of the ^89^Zr-FA-DFO-liposome and the ^89^Zr-DFO-liposome, the kidney, liver, bone, and tumor uptake at all three time points was extracted from the corresponding PET images and expressed as % ID/mL (Figure 3C and D). For both liposomes, the radioactivity was gradually washed out from the kidney, liver, and tumor, while the bone uptake increased over time. The ex vivo biodistributions of ^89^Zr-FA-DFO-liposome and ^89^Zr-DFO-liposome were measured in KB tumor xenograft-bearing CD1 nude mice at 48 h post-i.v. injection and compared with that of the free ^89^Zr-DFO (n=3) (Figure 4A and Table S2 present the biodistribution data). The kidney uptake of ^89^Zr-FA-DFO-liposome was about three-fold higher than the ^89^Zr-DFO-liposome. In contrast, the liver, spleen, and colon uptake of ^89^Zr-FA-DFO-liposome was two-, four-, and five-fold lower than the ^89^Zr-DFO-liposome, respectively. The tumor uptake of ^89^Zr-FA-DFO-liposome was two-and-a-half-fold lower than that of the ^89^Zr-DFO-liposome. To compare the relative uptake of tumor to nontarget organs, the uptake ratio of tumor to blood, muscle, and other internal organs for both ^89^Zr-FA-DFO-liposome and ^89^Zr-DFO-liposome was measured at 48 h post-i.v. injection and is illustrated in Figure 4B. Furthermore, in contrast to the ^89^Zr-labeled liposomes, the free ^89^Zr-DFO had little uptake in the tumor and other organs, apart from the kidney at 48 h post-i.v. injection. In addition, the biodistribution of the ^89^Zr-PEG-DFO-liposome containing three-fold higher PEG_2000_ than the other two liposomes was also investigated at 48 h post-i.v. injection in healthy CD1 mice (n=3) used as control (Figure 4A and Table S2 present the biodistribution data). Its biodistribution pattern was similar to the ^89^Zr-FA-DFO-liposome with increased kidney uptake and reduced liver and spleen uptake compared with the ^89^Zr-DFO-liposome.

## Discussion

Efficient liposome labeling with ^89^Zr at room temperature requires both the lipophilic weak zirconium chelator, 8-HQ, and the hydrophilic strong zirconium chelator, DFO, to be labeled quantitatively with ^89^Zr. In addition, the ligand exchange reaction between the ^89^Zr(8-HQ)_4_ complex and DFO, which must occur inside the liposome, should also be fast and quantitative. After systematically screening different ligand concentrations, buffers, pHs, and reaction times, we found that both the ^89^Zr(8-HQ)_4_ and ^89^Zr-DFO can be prepared in near-quantitative RCYs at room temperature in pH 7.0 HEPES buffer. The ligand exchange reaction between ^89^Zr(8-HQ)_4_ and DFO can also reach completion at room temperature when the DFO concentration is above 10 mM. Therefore, a thermosensitive DFO-liposome, an FA-decorated active targeting FA-DFO-liposome, and a PEG-DFO-liposome were prepared with DFO (10.0 mM) encapsulated in their aqueous cavity. The newly formed materials showed characteristics of liposomal nanoparticles in terms of their average particle size, PDI, and zeta potential when compared with those reported in the literature.^14^–^16^ Subsequently, we identified the ^89^Zr:8-HQ:DFO-liposome in a volume ratio of 2:1:6 containing approximately 4.0 mM of lipids as optimal ^89^Zr liposome-radiolabeling formulation, which offers near-quantitative RCYs for all three liposomal nanoparticles at room temperature. There was no membrane-bound ^89^Zr in the labeled liposomes, as confirmed by the DFO challenge experiment. We found that the concentration of the DFO-liposome is crucial for the completion of the ligand exchange reaction between the ^89^Zr(8-HQ)_4_ and the encapsulated DFO. When half of the DFO-liposome is used, the unreacted but encapsulated ^89^Zr(8-HQ)_4_ can slowly leak out of the liposome in 48 h. The excellent isolated RCYs and reproducibility of this radiolabeling method enabled us to produce sufficient ^89^Zr-labeled liposomes for preclinical PET imaging studies (three mice), starting with only around 20 MBq of ^89^Zr. Furthermore, as ^89^Zr has strong gamma emission above 909 keV, this highly efficient and rapid liposome-radiolabeling strategy is extremely beneficial to the radiochemists for minimizing their radiation dose by using minimal amount of ^89^Zr and minimizing its manipulation by avoiding the preparation of ^89^Zr(8-HQ)_4_ in a chloroform/carbonate buffer biphase system, separation of the ^89^Zr(8-HQ)_4_ containing organic phase, and followed by evaporating the chloroform and reformulating it in PBS for liposome labeling, as reported by Edmonds et al.^13^ This technically simple one-pot two-step method can be readily implemented on an automatic radiosynthesizer such as GE Fast Lab^®^ or Eckert & Ziegler Modular Lab^®^, which enables the good manufacturing practice preparation of ^89^Zr-labeled liposomes for clinical use. Furthermore, all the ^89^Zr-labeled liposomes showed excellent stability in PBS at 37°C for 48 h. The radiolabel retention of ^89^Zr-FA-DFO-liposome only decreased slightly to 94% in fresh rat serum at 37°C for 48 h indicating that the hydrophilic ^89^Zr-DFO cannot leak out of the lipid membrane under these conditions.

In the in vitro KB cell uptake study, the near 15-fold increased accumulation of ^89^Zr-FA-DFO-liposome in these folate receptor-overexpressing cells compared to the ^89^Zr-DFO-liposome demonstrated the active targeting property of this liposome. As a noninvasive imaging tool, PET can quantitatively measure the in vivo biodistribution and pharmacokinetics of the ^89^Zr-labeled liposomal nanoparticles. This information is critical for further optimizing their physical and biochemical properties as drug delivery vehicles.^17^ To demonstrate this application, we carried out three sequential PET imaging studies at 6, 24, and 48 h post-i.v. injection with both ^89^Zr-FA-DFO-liposome and ^89^Zr-DFO-liposome in the KB tumor xenograft-bearing CD1 nude mice. In the PET images, the KB tumor xenografts were visible at 6 h, and became clearer at 24 h because of the increased tumor-to-background contrast, and then the signals became weaker at 48 h due to washing out of radioactivity over time. Furthermore, uneven distribution of the radioactivity signals was observed in the tumor xenografts with both types of ^89^Zr-labeled liposomes due to the heterogeneity of these tumors. The subsequent pharmacokinetic analysis from the PET images indicated that the radioactivity was gradually washed out from the metabolizing organs such as kidney and liver and deposited in the bone over time. The biodistribution study at 48 h post-i.v. injection unveiled the detailed distribution and the metabolism of both ^89^Zr-FA-DFO-liposome and ^89^Zr-DFO-liposome. The biodistribution patterns of the two liposomes revealed significant differences. The ^89^Zr-FA-DFO-liposome was mainly accumulated in the kidney, whereas the uptake of ^89^Zr-DFO-liposome in the liver, spleen, and colon was much higher. This is most likely due to the higher expression of folate receptors in the kidney leading to increased renal accumulation of the ^89^Zr-FA-DFO-liposome and reducing its availability to other metabolizing organs. Surprisingly, the tumor uptake of ^89^Zr-FA-DFO-liposome was two-and-a-half-fold lower than that of the ^89^Zr-DFO-liposome, which is in great contrast to the KB cell uptake in the in vitro experiments. We speculated that this could be because of the overwhelming competitive accumulation of the ^89^Zr-FA-DFO-liposome in the kidneys reduced its availability to the tumor. These results suggest that the enhanced permeability and retention effect plays a greater part in retention of liposomes in the tumor than specific molecular targeting to folate receptor. It is further evidenced by the fact that both ^89^Zr-labeled liposomal nanoparticles exhibit significantly higher tumor uptake compared with the free ^89^Zr-DFO at this time point. It is worth noting that higher uptake ratio of tumor to blood, muscle, and other internal organs (but not bone, spleen, liver, or kidney) was observed for both ^89^Zr-FA-DFO-liposome and ^89^Zr-DFO-liposome which generated excellent target-to-background contrast to visualize the tumors and the ^89^Zr intratumor deposition. Significant ^89^Zr bone uptake was observed from both liposomes, whereas there was little bone uptake for the free ^89^Zr-DFO at 48 h. We envisage that the dissociation of the liposome-encapsulated ^89^Zr-DFO is likely taking place in the liver and other reticuloendothelial sites where initial uptake of liposomes is most likely. As a small hydrophilic molecule, the free ^89^Zr-DFO was rapidly excreted through the kidney with little chance for hepatic accumulation. In contrast, the liver uptake for the ^89^Zr-FA-DFO-liposome and ^89^Zr-DFO-liposome was three- and five-fold higher, respectively, than that of free ^89^Zr-DFO, which would result in greater hepatic catabolism of the liposome-encapsulated ^89^Zr-DFO causing release of ^89^Zr into the circulation, leading to its accumulation in bone. This hypothesis is also supported by the fact that the renal avid ^89^Zr-FA-DFO-liposome had four-fold less bone uptake than the ^89^Zr-DFO-liposome, likely because of its reduced liver and spleen uptake and hence reduced release of free ^89^Zr. In addition, the biodistribution of the renal avid ^89^Zr-PEG-DFO-liposome at 48 h is very similar to that of the ^89^Zr-FA-DFO-liposome due to its increased PEG content in the membrane. Because of its increased kidney uptake and reduced liver uptake, less ^89^Zr bone deposition for the ^89^Zr-PEG-DFO-liposome was observed compared to the ^89^Zr-DFO-liposome, despite lacking the active targeting shown by the ^89^Zr-FA-DFO-liposome. A weakness of the present liposome formulation is that it has poor resistance against endothelial uptake, which leads to catabolism of the liposomes and release of free ^89^Zr, as indicated by uptake in the skeleton which complicates the interpretation of overall biodistribution. Nevertheless, it is likely that the method can provide a good indication of quantitative delivery to the tumor, which is the main potential clinical application of a system such as this.

## Conclusion

We developed a highly efficient ^89^Zr liposome-labeling method based on a rapid ligand exchange reaction. This novel method is technically simple and does not require lipid modification. It is particularly suitable for ^89^Zr labeling of thermosensitive liposomal nanoparticles as it can achieve near-quantitative isolated RCYs at room temperature. The ^89^Zr-labeled liposomes have excellent in vitro stability in PBS and rat serum at 37°C for 48 h. Sequential PET/CT scan together with the ex vivo biodistribution studies demonstrated the benefit of using the long-half-life ^89^Zr to determine the in vivo biodistribution, kinetics, and metabolism of the liposomal nanoparticles including the heterogeneous distribution of ^89^Zr-FA-DFO-liposome and ^89^Zr-DFO-liposome in the KB tumor xenografts, the extensive kidney accumulation of the ^89^Zr-FA-DFO-liposome and ^89^Zr-PEG-DFO-liposome, and the different metabolic fate of the free and liposome-encapsulated ^89^Zr-DFO. It also identified the weakness of the present liposome formulation as all three liposomes exhibited high endothelial uptake, which resulted in their catabolism and consequently release of free ^89^Zr. Thus, this new radiolabeling method can become a generic tool to guide the development and clinical application of the liposomal nanoparticles.

## Supplementary materials

Figure S1 HPLC chromatogram of crude reaction mixture of ^89^Zr and 8-HQ. ^89^Zr(8-HQ)_4_ with retention time of 16.6 min detected by radioactivity detector. HPLC chromatography from UV detector (**A**); HPLC chromatography from radioactivity detector (**B**).
**Abbreviations:** HPLC, high-performance liquid chromatography; 8-HQ, 8-hydroxyquinoline; UV, ultraviolet; UV-A, ultraviolet absorption; ChA, chromatography; CPS, counts per second; Reg, region.

Figure S2 HPLC chromatogram of crude reaction mixture of ^89^Zr and DFO. ^89^Zr-DFO with retention time of 16.4 min detected by radioactivity detector. HPLC chromatography from UV detector (**A**); HPLC chromatography from radioactivity detector (**B**).
**Abbreviations:** HPLC, high-performance liquid chromatography; DFO, deferoxamine; UV, ultraviolet; UV-A, ultraviolet absorption; ChA, chromatography; CPS, counts per second.

Figure S3 RadioTLC of free ^89^Zr, ^89^Zr-DFO, and ^89^Zr(8-HQ)_4_.
**Abbreviations:** TLC, thin-layer chromatography; DFO, deferoxamine; 8-HQ, 8-hydroxyquinoline.

Figure S4 Size exclusion purification of ^89^Zr-DFO-liposome eluted in the fraction from 0.4 to 2.2 mL.
**Abbreviation:** DFO, deferoxamine.

Figure S5 KB cell uptake of ^89^Zr-FA-DFO-liposome and ^89^Zr-DFO-liposome in vitro. The results are presented as % incubation dose per million cells.
**Abbreviations:** FA, folic acid; DFO, deferoxamine.

**Table S1:** Characterization of a thermosensitive DFO-liposome, an FA-decorated active targeting FA-DFO-liposome, and a PEG-DFO-liposome: mean size, PDI, and zeta potential

Samples	Size (nm)	PDI	Zeta potential (mV)
DFO-liposome	99.8±0.9	0.120	−22.2
FA-DFO-liposome	102.9±2.3	0.110	−20.1
PEG-DFO-liposome	101.2±3.1	0.126	−23.1

**Note:** Values are presented as the mean ± standard deviation (n=3).

**Abbreviations:** DFO, deferoxamine; FA, folic acid; PEG, polyethylene glycol; PDI, polydispersity index.

**Table S2:** Biodistribution of ^89^Zr-FA-DFO-liposome, ^89^Zr-DFO-liposome, and ^89^Zr-DFO in KB tumor xenograft-bearing CD1 nude mice (n=3) at 48 h post-i.v. injection and ^89^Zr-PEG-DFO-liposome in healthy CD1 mice (n=3) at 48 h post-i.v. injection expressed as % ID/g

Organ	^89^Zr-FA-DFO-liposome (n=3)	^89^Zr-DFO-liposome (n=3)	^89^Zr-DFO (n=3)	^89^Zr-PEG-DFO-liposome (n=3)
Blood	0.087±0.103	0.146±0.079	N/A	0.137±0.120
Liver	0.570±0.226	1.083±0.559	0.201±0.054	0.520±0.162
Heart	0.093±0.073	0.215±0.099	0.018±0.005	0.156±0.090
Kidney	3.926±2.898	1.321±0.117	2.054±0.275	4.872±2.161
Colon	0.105±0.065	0.526±0.700	0.204±0.154	0.309±0.206
Lung	0.203±0.142	0.328±0.112	0.209±0.231	0.142±0.067
Muscle	0.051±0.042	0.093±0.037	0.058±0.037	0.063±0.018
Spleen	0.377±0.227	1.639±0.141	0.050±0.006	0.445±0.262
Stomach	0.120±0.092	0.203±0.090	0.169±0.078	0.391±0.311
Bone	2.516±1.704	10.857±5.283	0.121±0.035	2.198±0.473
Small intestine	0.105±0.067	0.144±0.055	0.126±0.073	0.283±0.191
Tumor	0.588±0.518	1.533±0.552	0.069±0.019	N/A

**Note:** Data presented as mean ± standard deviation.

**Abbreviations:** FA, folic acid; DFO, deferoxamine; i.v., intravenous; PEG, polyethylene glycol; ID, injected dose; N/A, not available.

## Figures and Tables

**Table 1:** Optimization of ^89^Zr radiolabeling of liposomes

Entry^a^	Lipophilic ligand^b^	Liposome (300 µL)	Isolated RCYs
1^c^	8-HQ	DFO-liposome	98%±2% (n=3)
2	8-HQ	DFO-liposome	98%±2% (n=5)
3	8-HQ	FA-DFO-liposome	98%±1% (n=6)
4	8-HQ	PEG-DFO-liposome	97%±1% (n=3)
5^c^	8-HQ	Blank liposome	22%
6	N/A	DFO-liposome	81%±11% (n=3)
7	2-HQ	DFO-liposome	83%

**Notes:**

a ^89^Zr (5.0 MBq) in HEPES buffer (100 µL, 1.0 M, pH 7.0).

b PBS (50 µL) for entry 6, or 2-HQ or 8-HQ (23 µg, 0.16 µmol) in HEPES buffer (50 µL, 10 mM, pH 7.0) for the rest of the experiments.

c One hundred and fifty microliters of the corresponding liposomes were used. Data presented as mean ± standard deviation.

**Abbreviations:** RCYs, radiochemical yields; 8-HQ, 8-hydroxyquinoline; DFO, deferoxamine; FA, folic acid; PEG, polyethylene glycol; N/A, not available; 2-HQ, 2-hydroxyquinoline; HEPES, 4-(2-hydroxyethyl)-1-piperazineethanesulfonic acid; PBS, phosphate-buffered saline.

## Abbreviations

CT: computed tomography
DFO: deferoxamine
DPPC: 1,2-dipalmitoyl-sn-glycero-3-phosphocholine
DSPE: 1,2-distearoyl-sn-glycero-3-phosphoethanolamine
DSPE-PEG_2000_: 1,2-distearoyl-sn-glycero-3-phosphoethanola mine-*N*-[methoxy(polyethylene glycol)-2000]
DSPE-PEG_2000_ Folate: 1,2-distearoyl-sn-glycero-3-phosphoethanolamine-*N*-[folate(polyethylene glycol)-2000]
DTPA: diethylenetriaminepentaacetic acid
FA: folic acid
HEPES: 4-(2-hydroxyethyl)-1-piperazineethanesulfonic acid
HPLC: high-performance liquid chromatography
2-HQ: 2-hydroxyquinoline
8-HQ: 8-hydroxyquinoline
ID: incubation dose
i.v.: intravenous
PBS: phosphate-buffered saline
PDI: polydispersity index
PEG: polyethylene glycol
PEG1k: (polyethylene glycol)-1000
PET: positron emission tomography
RCYs: radiochemical yields
Rf: retardation factor
TEM: transmission electron microscopy
TFA: trifluoroacetic acid
TLC: thin-layer chromatography

## References

[1] Fenske DB, Chonn A, Cullis PR (2008). Liposomal nanomedicines: an emerging field. Toxicol Pathol.

[2] Ta T, Porter TM (2013). Thermosensitive liposomes for localized delivery and triggered release of chemotherapy. J Control Release.

[3] Noble GT, Stefanick JF, Ashley JD, Kiziltepe T, Bilgicer B (2014). Ligand-targeted liposome design: challenges and fundamental considerations. Trends Biotechnol.

[4] McGranahan N, Swanton C (2015). Biological and therapeutic impact of intratumor heterogeneity in cancer evolution. Cancer Cell.

[5] Gambhir SS (2002). Molecular imaging of cancer with positron emission tomography. Nat Rev Cancer.

[6] Emmetiere F, Irwin C, Viola-Villegas NT (2013). ^18^F-labeled-bioorthogonal liposomes for in vivo targeting. Bioconjug Chem.

[7] Petersen AL, Binderup T, Rasmussen P (2011). ^64^Cu loaded liposomes as positron emission tomography imaging agents. Biomaterials.

[8] Abou DS, Thorek DL, Ramos NN (2013). ^89^Zr-labeled paramagnetic octreotide-liposomes for PET-MR imaging of cancer. Pharm Res.

[9] Seo JW, Mahakian LM, Tam S (2015). The pharmacokinetics of Zr-89 labeled liposomes over extended periods in a murine tumor model. Nucl Med Biol.

[10] Pérez-Medina C, Abdel-Atti D, Zhang Y (2014). A modular labeling strategy for in vivo PET and near-infrared fluorescence imaging of nanoparticle tumor targeting. J Nucl Med.

[11] Ferris TJ, Charoenphun P, Meszaros LK, Mullen GED, Blower PJ, Went MJ (2014). Synthesis and characterisation of zirconium complexes for cell tracking with Zr-89 by positron emission tomography. Dalton Trans.

[12] Charoenphun P, Meszaros LK, Chuamsaamarkkee K (2015). [^89^Zr] Oxinate_4_ for long-term in vivo cell tracking by positron emission tomography. Eur J Nucl Med Mol Imaging.

[13] Edmonds S, Volpe A, Shmeeda H (2016). Exploiting the metal-chelating properties of the drug cargo for *in vivo* positron emission tomography imaging of liposomal nanomedicines. ACS Nano.

[14] Tan X, Pang X, Lei M (2016). An efficient dual-loaded multifunctional nanocarrier for combined photothermal and photodynamic therapy based on copper sulfide and chlorin e6. Int J Pharm.

[15] Guo F, Yu M, Wang J, Tan F, Li N (2015). Smart IR780 theranostic nanocarrier for tumor-specific therapy: hyperthermia-mediated bubble-generating and folate-targeted liposomes. ACS Appl Mater Interfaces.

[16] Marik J, Tartis MS, Zhang H (2007). Long-circulating liposomes radiolabeled with [^18^F]fluorodipalmitin ([^18^F]FDP). Nucl Med Biol.

[17] Drummond DC, Noble CO, Hayes ME, Park JW, Kirpotin DB (2008). Pharmacokinetics and in vivo drug release rates in liposomal nanocarrier development. J Pharm Sci.

