# Supplementary figures and images for: A generic 89Zr labeling method to quantify the in vivo pharmacokinetics of liposomal nanoparticles with positron emission tomography
Source: Int J Nanomedicine. 2017 Apr 20;12:3281–94. doi: 10.2147/IJN.S134379 (PMC5404495; doi:10.2147/IJN.S134379)

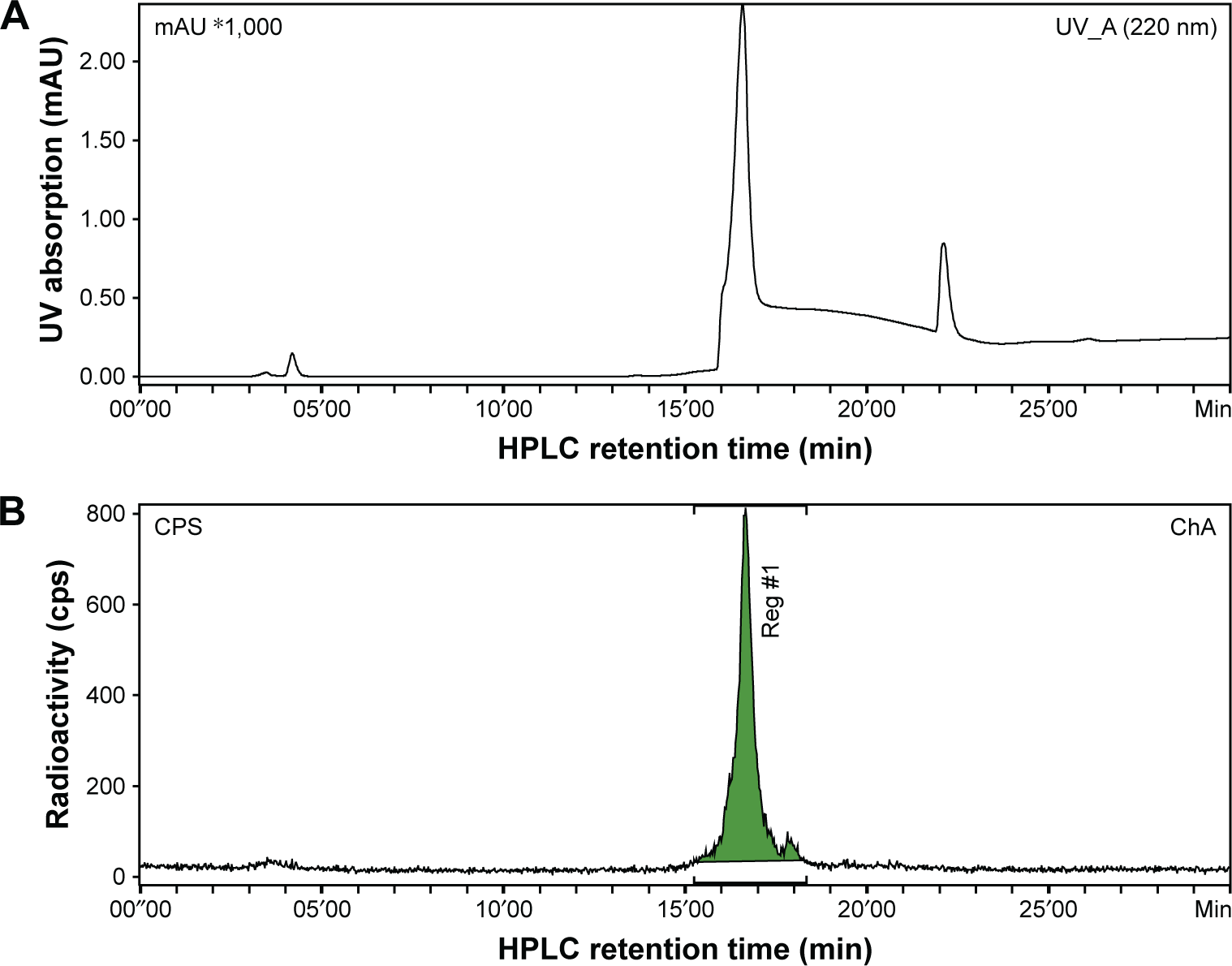

Supplement: Figure S1 — HPLC chromatogram of crude reaction mixture of 89Zr and 8-HQ. 89Zr(8-HQ)4 with retention time of 16.6 min detected by radioactivity detector. HPLC chromatography from UV detector (A); HPLC chromatography from radioactivity detector (B). Abbreviations: HPLC, high-performance liquid chromatography; 8-HQ, 8-hydroxyquinoline; UV, ultraviolet; UV-A, ultraviolet absorption; ChA, chromatography; CPS, counts per second; Reg, region. [file ijn-12-3281s1.tif]

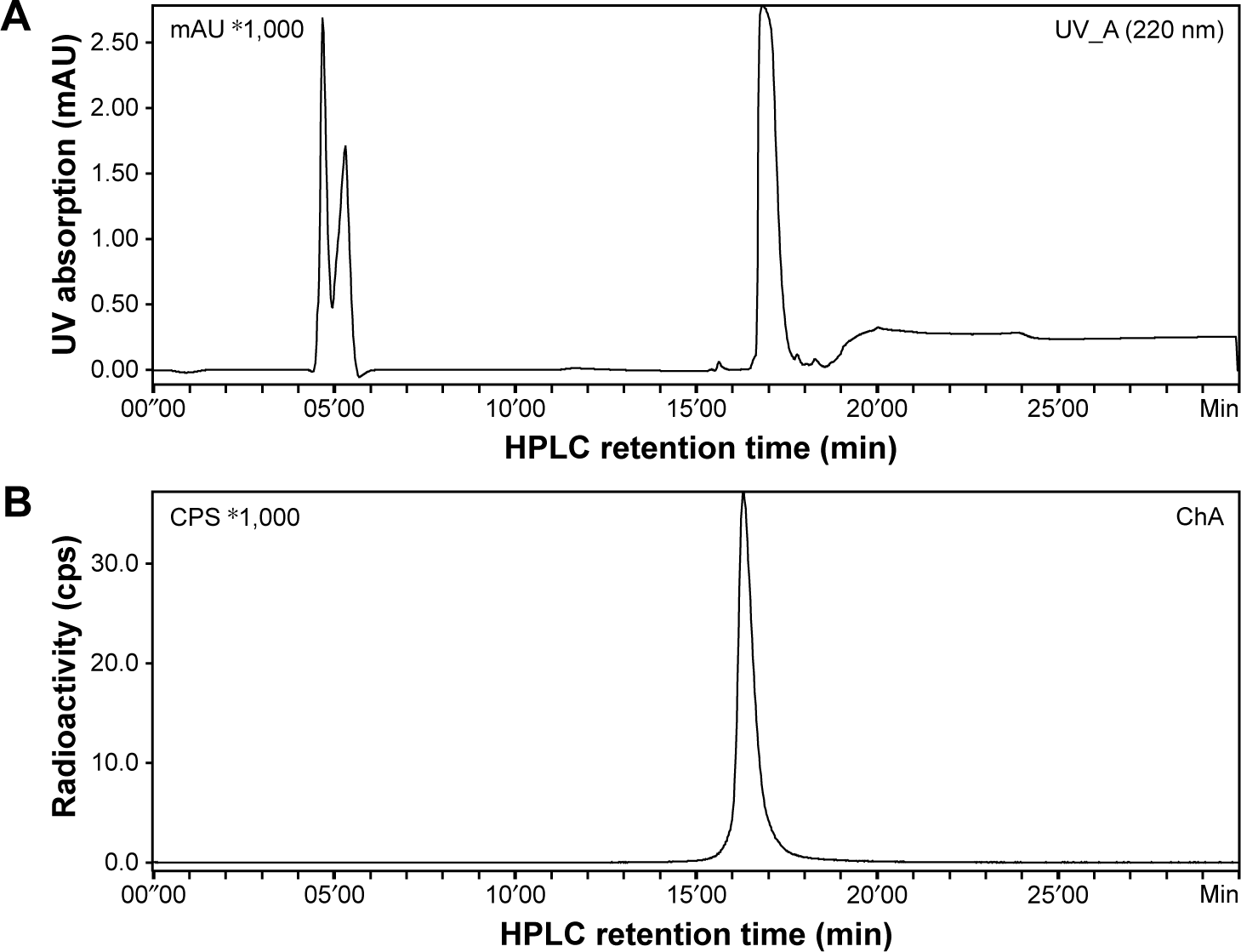

Supplement: Figure S2 — HPLC chromatogram of crude reaction mixture of 89Zr and DFO. 89Zr-DFO with retention time of 16.4 min detected by radioactivity detector. HPLC chromatography from UV detector (A); HPLC chromatography from radioactivity detector (B). Abbreviations: HPLC, high-performance liquid chromatography; DFO, deferoxamine; UV, ultraviolet; UV-A, ultraviolet absorption; ChA, chromatography; CPS, counts per second. [file ijn-12-3281s2.tif]

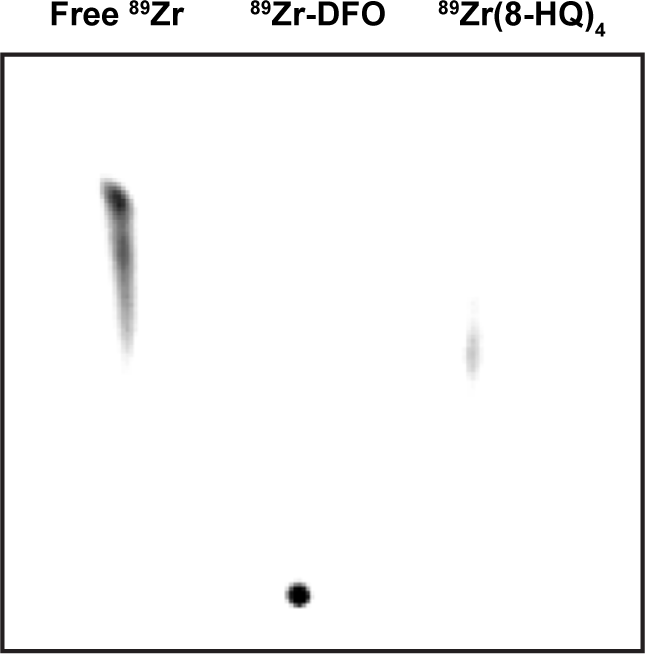

Supplement: Figure S3 — RadioTLC of free 89Zr, 89Zr-DFO, and 89Zr(8-HQ)4. Abbreviations: TLC, thin-layer chromatography; DFO, deferoxamine; 8-HQ, 8-hydroxyquinoline. [file ijn-12-3281s3.tif]

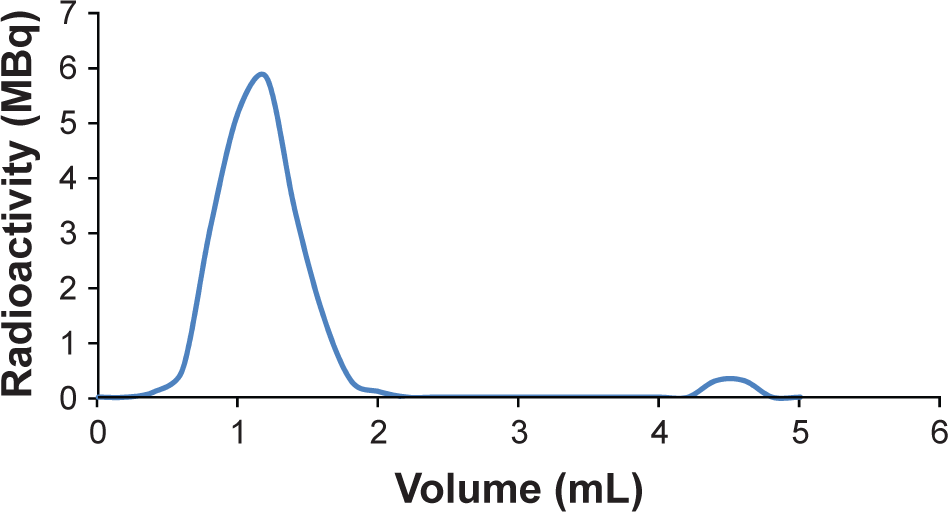

Supplement: Figure S4 — Size exclusion purification of 89Zr-DFO-liposome eluted in the fraction from 0.4 to 2.2 mL. Abbreviation: DFO, deferoxamine. [file ijn-12-3281s4.tif]

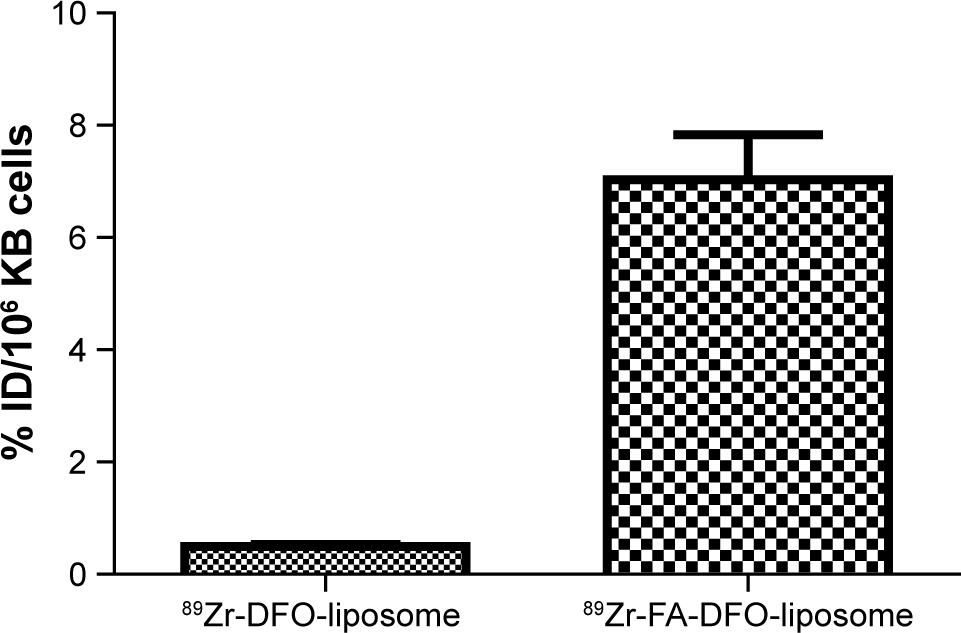

Supplement: Figure S5 — KB cell uptake of 89Zr-FA-DFO-liposome and 89Zr-DFO-liposome in vitro. The results are presented as % incubation dose per million cells. Abbreviations: FA, folic acid; DFO, deferoxamine. [file ijn-12-3281s5.tif]
